# Honey bee hive covers reduce food consumption and colony mortality during overwintering

**DOI:** 10.1371/journal.pone.0266219

**Published:** 2022-04-04

**Authors:** Ashley L. St. Clair, Nathanael J. Beach, Adam G. Dolezal

**Affiliations:** 1 Department of Entomology, University of Illinois Urbana-Champaign, Urbana, Illinois, United States of America; 2 Carl R. Woese Institute for Genomic Biology, University of Illinois at Urbana-Champaign, Urbana, Illinois, United States of America; King Khalid University, SAUDI ARABIA

## Abstract

Beekeepers regularly employ management practices to mitigate losses during the winter, often considered the most difficult time during a colony life cycle. Management recommendations involving covering or wrapping hives in insulation during winter have a long history; over 100 years ago, most recommendations for overwintering in cold climates involved heavy insulation wraps or moving hives indoors. These recommendations began to change in the mid-20^th^ century, but hive covers are still considered useful and are described in contemporary beekeeping manuals and cooperative extension materials. However, most of the data supporting their use is published primarily in non-peer reviewed trade journals and was collected >40 years ago. In this time, the beekeeping environment has changed substantially, with new pressures from pathogens, agrochemicals, and land use changes. Here, we provide an update to the historical literature, reporting a randomized experiment testing the effectiveness of a common honey bee hive cover system across eight apiaries in central Illinois, USA, a temperate region dominated by conventional annual agriculture. We found that, when other recommended overwintering preparations are performed, covered colonies consumed less food stores and survived better than uncovered controls (22.5% higher survival). This study highlights the value of hive covers, even in an area not subject to extremely cold winter conditions, and these data can aid the production of evidence-based extension recommendations for beekeepers.

## Introduction

Over the last several decades, worldwide interest in beekeeping has grown [1–4] while operational colony losses have remained high or increased [5]. For example, in the winter of 2018–19, beekeepers across the E.U. reported average winter losses of 16.7% and as high as 32% in some countries (e.g., Slovenia) [6]. Over the winter of 2020–21, U.S. beekeepers reported average winter losses of 32.2%, with some states reporting losses as high as 58% [7]. These loss rates are higher than historical estimates in the U.S., which were under 20% [8], and estimates from the E.U. which were 10–12% [9–11]. Although the threshold for acceptable rates of loss has been steadily increasing in recent years, likely as an influence of media and beekeepers becoming accustomed to increased losses [7, 12], winter mortality in the U.S. remains higher than what beekeepers report as an acceptable loss rate of 23.3% [7]. To reduce these losses, beekeepers continue to employ a variety of management practices depending on their skill level, operation size, climate, production goals, and beekeeping philosophy [13, 14]. Among these, wrapping or covering hives with extra material during winters has received renewed interest and use among many beekeepers and is often recommended in extension materials [15].

Temperate winters are hallmarked by cold temperatures and a cessation of forage (i.e., floral resource) availability for bees [16, 17]. Honey bees do not hibernate; rather, a cluster of worker bees feed upon their accumulated food stores, fueling heat production to thermoregulate and maintain a stable temperature throughout the season [18–22]. Because bees are mostly restricted to a nest with finite food stores, winter is usually considered the highest risk period for colony survival [16]. As such, there is a long history of beekeepers employing management strategies to improve overwinter survival for their colonies [23]. From at least the early 20^th^ century, beekeepers in cold climates commonly covered hives with wooden sleeves or wooly insulation material, partially buried hives in trenches, and kept hives inside cellars. In popular beekeeping manuals of the time, it was even recommended to forgo uncovered, outdoor overwintering completely [23, 24]. For example, it was recommended that beekeepers must overwinter indoors or with wrappings if they were located north of 42°N (approximately Chicago, IL and Boston, MA in the USA), and outdoor overwintering was only recommended south of 40°N (approximately Indianapolis, IN and Philadelphia, PA in the USA). [24].

Starting in the 1940s, Farrar performed a series of experiments showing that winter clusters in hives kept near Madison, WI, USA (approximately 43°N) can efficiently thermoregulate outdoors without extra hive coverings [18–20]. Soon after, Simpson further explained how clustering honey bees achieve nest thermoregulation [21]. Thus, recommendations began to shift, and beekeepers were more likely to overwinter colonies outdoors without heavy wrappings [23, 25, 26]. However, hive covers have continued to be recommended in beekeeping texts and extension documents, especially in those targeted at beekeeping in “cold” or “northern” climates [15, 27], though the exact definitions of these ranges are often not explicitly stated. It is still not clear how necessary or valuable wrappings are in many cases, especially in areas where winters are not extremely cold [28].

While many basic beekeeping practices have remained similar since these foundational studies, dramatic changes have also occurred [20, 25], including the introduction of new pests, parasites, and pathogens [29], shifts in land use that impact bee productivity [17, 30–32], and changes in pesticide stress and exposure [33, 34]. Due to these factors, wintering has become even more perilous, with managed honey bee colony losses posing major challenges for modern beekeepers [7]. Thus, hive coverings or wraps remain appealing to many beekeepers as a management practice for aiding colony overwintering. Despite this, there is a surprising paucity of published scientific research on the effects and efficacy of hive covers, especially from the 21^st^ century. While historical trials and data on wraps exists, they are often published in trade journals (e.g., [18, 19, 26] and are thus not part of the peer-reviewed scientific record; examples in S1 Table). What historical literature does exist on hive wrapping is primarily focused on use in very cold climates (e.g., Wisconsin and Minnesota), which may not be widely applicable, as climate change has resulted in significant temperature shifts since the foundational work in the 1940s and 1950s. In Illinois, where our study was performed, temperature increases have been particularly dramatic during winter and spring, with higher average temperatures and fewer nights where temperatures are below freezing [35]. Thus, if winter wrapping is primarily beneficial in colder climates, one might expect hive covers to be less effective than >50 years ago. Some recent work has shown that different hive construction materials (i.e., wooden vs polyurethane hive bodies), without any additional wrapping, can reduce temperature and humidity fluctuations but has not connected to overwintering survival or consumption of food stores [36].

Here, we sought to evaluate the benefits of covering outdoor honey bee hives with corrugated polypropylene sheets with insulation foam tops. Similar waterproof corrugated material has been used for hive covers since at least the 1960s [27], replacing tarpaper or other building paper as a windbreak wrap [20], and remains easily found through beekeeping suppliers today. However, to our knowledge, little peer-reviewed work exists evaluating the effectiveness of using these covers to improve honey bee colony thermoregulation, reduce food consumption, and improve overwintering survival. We hypothesized that insulating hives with polypropylene hive covers and top insulators would help buffer them from temperature fluctuations and allow bees to heat the winter cluster more efficiently, ultimately reducing food consumption required to maintain thermoregulation, improving survival, and increasing spring colony growth. To test this, we ran a randomized trial of 43 hives across 8 apiaries through an overwintering season in a temperate region dominated by annual agriculture.

## Methods

### Bee source and study sites

**Fig 1:**
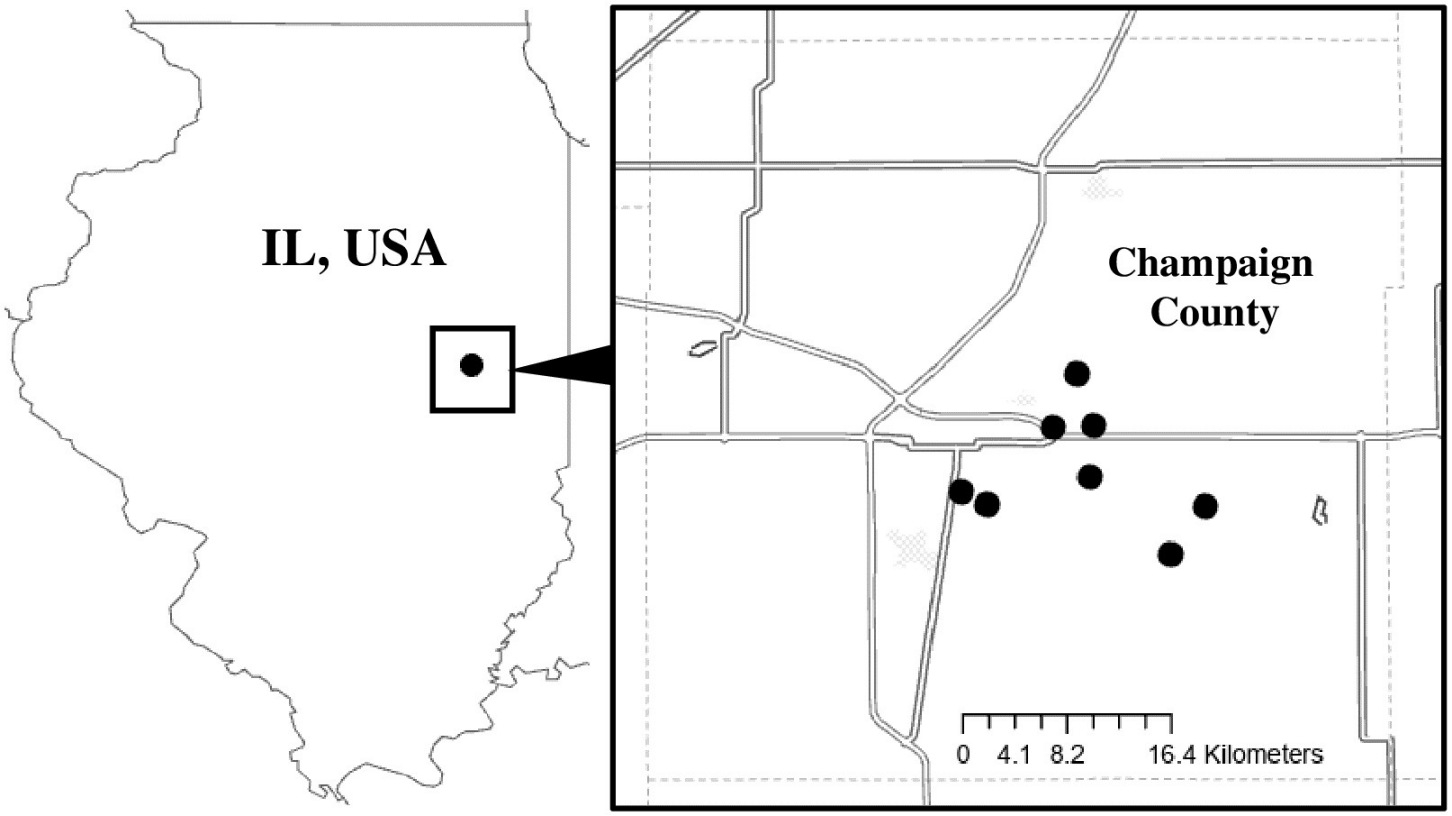
Location of apiaries in Champaign county Illinois, USA over the winter of 2020–2021.

For this study, we used 43 full-sized honey bee colonies kept in standard 10-frame Langstroth hive bodies managed by the University of Illinois Bee Research Facility in Urbana, Illinois, USA (located at approximately 40°N) during 2020. Of these 22 were in the covered treatment group and 21 in the uncovered. All colonies were headed by a commercially sourced Carniolan (*Apis mellifera carnica*) or Italian (*A*. *m*. *ligustica*) honey bee queen or her direct descendant. Hives were kept across eight apiaries within Champaign County, with each apiary housing an average of 5.5 (±0.85 SEM) hives (Fig 1).

### Pre-overwintering management

On a monthly basis, we performed routine alcohol washes of approximately 300 nurse aged bees to monitor the population of the parasitic mite *Varroa destructor* within all colonies [37]. All the source colonies in this study were treated for Varroa mites in early August, before equalization, using amitraz strips, Apivar (Mann Lake ltd.), per label instructions In October, mite levels varied significantly across the apiaries and were higher than the suggested threshold of 1% (mean 10.54 mites/300 bees (3.5% infestation) S1A Fig; [37]), therefore, we treated all colonies using oxalic vaporization (OxaVap ProVap 110) per label instructions on October 12^th^. Mite levels were rechecked on November 9^th^ and found to be reduced below threshold (mean of 2.2 mites/300 bees (0.7% infestation), S1B Fig). At this time, there were no significant differences in mite load between colonies from the different, randomly selected, covered versus control treatment groups. A final oxalic acid vaporization treatment was performed on all colonies on January 6^th^. In all cases of mite treatments, every colony was treated identically, regardless of its mite load. On October 5^th^–8^th^, all colonies were condensed into two 10-frame deep hive bodies and a single 10 frame medium honey super filled with capped honey from the summer 2020. At that same time, all colonies then received 1 gallon (3.79 liters) of liquid honey derived from our beekeeping operation, then two weeks later (October 23^rd^) received 1 gallon (3.79 liters) of 2:1 sucrose solution; both were delivered in 1 gallon division board feeders (Dadant and Sons. Inc., Hamilton IL). To ensure each colony possessed the necessary food stores for a successful overwinter, using a threshold of 30 kg of stored honey (per recommendations from [38]), we measured the pre-overwintering weight on November 9^th^. All colonies were significantly heavier than this threshold (T_7_ = 15.42, *p*<0.0001), with an average weight by apiary of 63.62kg (±1.39kg SEM) (S2 Fig). There were significant differences in average colony mass between some apiaries (S2 Fig; F_7,34_ = 2.56, *p* = 0.03), but there were no differences between the colonies that would later form the covered and control treatment groups (T_14_ = 1.40, *p* = 0.18).

### Wrapping and insulation

**Fig 2:**
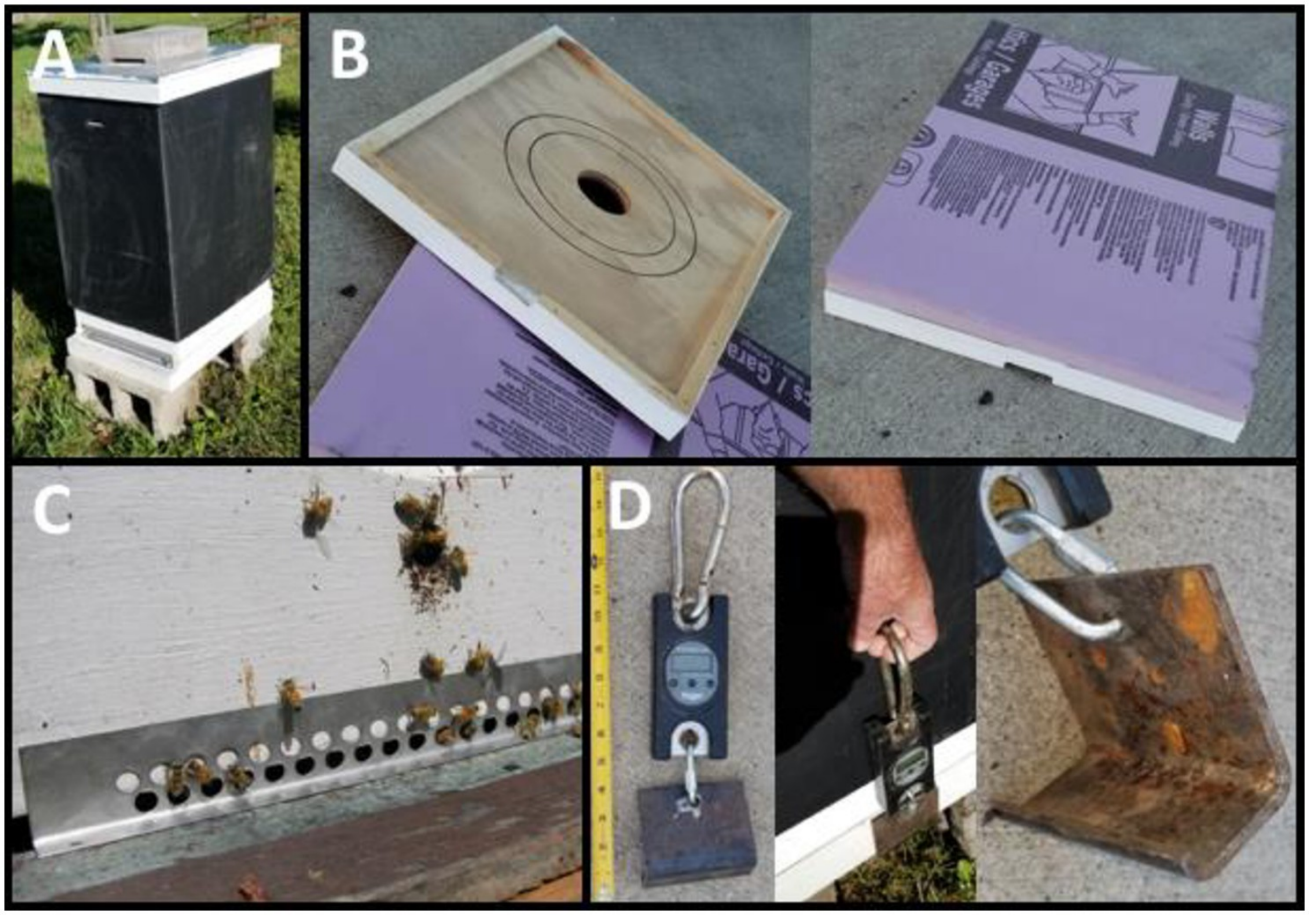
(A) Polypropylene hive cover on a honey bee colony. (B) Inner cover board and insulation that are placed on the top of the hive under the polypropylene cover. (C) Mouse guard on entrance to reduce rodent infestations. (D) Modified postal scale used to weigh colonies.

On November 12^th^, after colonies were condensed and supplementally fed, we randomly selected half of the focal colonies at each apiary to be placed into each treatment group (covered or control). Thus, at each apiary, we balanced the number of covered and control colonies when possible, resulting in a total of 22 covered and 21 uncovered controls across sites (S2 Table). Hive covers consisted of 4mm thick black corrugated polypropylene plastic sheets (Packaging Corporation of America in Conrad, IA) that were formed into rectangular prisms to slide over the top of the entire hive (Fig 2A), like those sold by Carters Honey Farms (https://www.cartershoneybees.com/product-page/overwinter-bee-hive-protective-cover). We placed 1.5-inch (3.81cm) foam insulation board (Owens Corning Foamular 250, R-7.5) on top of the inner covers of covered hives and top entrance holes were cut into each of the wraps to reduce condensation build up inside hives (Fig 2B). Entrance reducers and mouse guards were placed onto all colonies (covered and control) (Fig 2C).

### Mid-winter supplementation

To investigate whether colonies with covers consume a varied amount of mid-winter supplementation compared to uncovered control colonies, we placed a sugar cake patty that consisted of 7lbs. (3.18kg) dry granulated white sugar mixed with 1 cup (236.59 milliliters) of water in every colony. Sugar cakes were placed in the colonies on February 2^nd^ when the external temperature was greater than 45°F (7.25°C) to reduce the risk of chilling bees within the colonies. Sugar cakes were placed in the colonies using a 1-inch (2.54cm) shim lined with 27 gauge 1/8^th^ inch (0.32cm) wire mesh hardware cloth and were placed at the top of the colony above the honey super (S3 Fig). The sugar cake is very solid, and little or no sugar passively falls into the hive from it. To place the shim on colonies we removed the inner cover board. To account for the change in mass of equipment added and removed during the mid-winter supplementation all subsequent colony mass checks (see mass monitoring below) were adjusted as follows, ((current mass—mass of removed cover board) + mass of shim). This adjustment did not account for the 7lbs of added sugar cake as that weight would be variable across colonies as they consume the feed; however, this addition was consistent across all colonies when added on February 2^nd^. At the end of the wintering period when the first spring inspection was conducted (March 30^th^) the remaining sugar cake was removed from each colony after mass checks were recorded, brought back to the lab, dehydrated in a laboratory drying oven for 12 hours, and the mass of the remaining granulated sugar quantified.

### Temperature monitoring

To monitor the internal temperature of colonies, we placed a single thermocron iButton temperature meter (ibuttonlink.com) in each colony on November 9^th^. Temperature meters were placed between a strip of clear tape and inserted between two frames at the center of the uppermost deep sized hive body directly below the honey super. In addition to experimental colonies, we placed an iButton in one sentinel hive at three apiaries (HII, PT, and PF; total of 3 sentinel hives) to capture the variation in the ambient temperature compared to colony thermoregulatory temperatures. Sentinel hives consisted of a single Langstroth deep filled with 10 frames of drawn comb but empty of bees. The iButtons were set to record temperature within colonies once every 256 minutes from Nov. 9^th^ through March 30^th^. Because temperatures varied across the course of a day, we calculated the average temperature of a colony per calendar week to use in our analysis.

### Survivorship monitoring

To track colony survivorship throughout the winter, we used a medical stethoscope on the side of the hive body to listen for an audible buzz within. If no buzzing was heard, we used one sharp knock to attempt hearing another buzz. If no sounds were detected within a colony, that colony was considered dead for that timepoint. If, at the next timepoint that colony audibly buzzed then it would be considered alive at all previous timepoints. Survivorship checks were conducted in this way starting on November 9^th^ and continued every other week until March 30^th^, when we performed our first spring inspection of colonies, and all colonies were opened, and survivorship confirmed.

### Mass monitoring

To monitor the estimated consumption of honey stores within colonies over the winter, we tracked the mass of each colony starting on November 9^th^ and continuing every other week until March 30^th^. We used an industrial crane scale (SAGA Perseus) with a handle attached to one end and a lever which hooks to the underside of the colony on the opposite end (Fig 2D). We weighed the colony by lifting each side of the colony three times, taking an average weight of each side, and adding them together for total colony weight. Weighing the two sides of a colony with the lifting scale produces an imperfect biological mass, as all equipment is included and the position of the cluster can vary, however, within colony measurements are accurate and repeatable over time allowing for reliable measurements of change in mass. The scale features a “peak hold” setting which records and holds the maximum mass allowing the user to tilt the scale past center weight and obtain an accurate maximum weight for each side, this in combination with the average of three measurements per colony side reduces variation in weight due to user error. From this, we calculated the percent change in overwintering colony mass by subtracting the mass at each sample date from the starting mass of the colony on Nov. 9^th^, 2020. Specifically, percent mass was calculated as (1 ‒ (current mass/initial mass) *100). The rate of mass decline was also determined for each colony using the slope of the linear trend equation for an individual colony change in mass over time (kg mass decline per sampling period).

The crane scale method to weigh hives is critical for this study, as other methods of weighing colonies usually require opening and disassembling hive equipment to weigh them independently e.g., [17, 39], which would cause extreme stress during winter. We validated the crane scale weighing method by comparing the masses of 42 independent honey bee colonies, in the field during normal summer conditions. Each hive was measured with the crane scale method as described above and then by fully disassembling the hives and weighing the component pieces independently, as in Dolezal et al 2019 [17]. Using linear regression, we found that the crane scale method significantly predicted the weight determined by the more invasive method (F_1, 40_ = 136.7, *p* = <0.0001; S4 Fig), and the values were strongly correlated R^2^ = 0.77.

### Frames sides of bees and capped brood area

Adult bee populations in colonies during spring buildup were estimated twice; on March 30^th^ and April 12^th^. Populations were based on fractional estimates of sides of a frame covered in bees (i.e., “frame sides”) and capped brood area was estimated in each colony via photography following methods from [40]. In short, each frame was photographed, the area covered with brood was traced out using Photoshop, and the proportion pixels associated with capped brood was calculated. The proportion brood area was then converted to cm^2^ by multiplying the brood area by the area of the frame in cm^2^.

### Collection of bee samples and lipid analysis

To measure colony lipid levels of nurse bees at the end of the experiment, we collected approximately 50 bees from a frame containing open brood during our spring inspection (March 30^th^, 2020). Bees were transported to the lab on ice and immediately stored at -80°C until further processing. Bees were processed via the protocol of Toth and Robinson [41] as modified in Dolezal et al. [42]. Approximately 15 nurse bees, by mass, were homogenized in liquid nitrogen, and approximately 0.3g of homogenate was subsampled and weighed. Lipid content was quantified via phosphor-vanilin spectrophotometric assay and lipid calculated as mg lipid/mg bee mass.

### Landscape classification

We calculated the percent arboreal cover within 100m of colonies to better understand at what level variation in windbreak provided by tree cover contributed to the change in mass of colonies in covered vs control treatments. To calculate arboreal cover, we used Google Earth Pro to create a 100m radius buffer around the location of the colonies and then classified the number of pixels associated with the buffer in Photoshop. We then used Photoshop to create a layer that consisted of the arboreal cover that was within the buffer, calculated the pixels associated with this layer, and took a proportion of the total buffer.

Landscape scale land use surrounding each farm was quantified in ArcGIS, ArcMap 10.3.1 using a 1-km radius centered on the apiary location. Land use features were based on the US Department of Agriculture–National Agricultural Statistics Service cropland data layer for 2020 at a 30m × 30m resolution (https://nassgeodata.gmu.edu/CropScape/). Using the ‘histogram’ function in ArcMap, the proportion of all landscape feature classes were identified by counting pixels associated with each land category within the buffer (S3 Table). Land-use types were combined and categorized into four groups (cropland, developed, grassland, and woodland; S5 Fig).

### Statistical analysis

To compare colony temperatures across treatments, we created a mixed model analysis of variance in SAS using the ‘PROC GLIMMIX’ function. To meet the assumptions of normality, we used the natural log of the recorded temperature plus twenty-five as the response variable. Treatment (i.e., control vs covered), sample date (i.e., calendar week for temperature), and their interaction were fixed effects in the model. Colony nested within site was used as a random factor. If significant main effects or an interaction were observed, then we conducted a post hoc analysis of least squared means with Tukey HSD adjustment for multiple comparisons to look for differences between treatments on individual calendar weeks. Colonies that died over the winter were censored from the analysis at the time point death was recorded.

We used the same model listed above to assess percent change in mass (i.e., change in mass from Nov. 9^th^, 2020), proportion mid-winter supplement consumed, frame sides of bees, capped brood area, and colony lipid percent. Any colonies that died, were censored from the analysis of percent change in mass at their time of death. Rate of mass decline was assessed using the same model as above with the exception that the fixed effect of sample date was replaced with apiary. Analysis of rate of mass decline only included colonies that survived the entire overwintering season.

To better understand the relationship between land use in the surrounding landscape and colony mass decline we conducted a model selection using multiple regressions with stepwise model selection in SAS with function ‘PROC REG’. Land use types cropland, woodland, grassland, and developed land were included in our model selections and required a *p*-value <0.15 for model inclusion [43]. Because land cover types are inherently related to each other, we first ran a Pearson’s correlation using function ‘PROC COR’ in SAS to ensure that there were no collinearities among variables (Pearson’s correlation coefficient <0.8; S4 Table). To determine if there was a relationship with the percent arboreal cover within 100m of the apiary with the rate at which colonies lost mass we performed a linear regression in SAS using the ‘PROC REG’ function with rate of mass decline as the predictor variable and percent arboreal cover as the fixed effect. This analysis was performed for the covered and control treatments as well as at the apiary level.

To determine if colony overwintering survivability varied between treatments, we performed a Cox Hazard test in R (R Core Team 2019) using the ‘coxme’ package and function [44].

## Results

**Fig 3:**
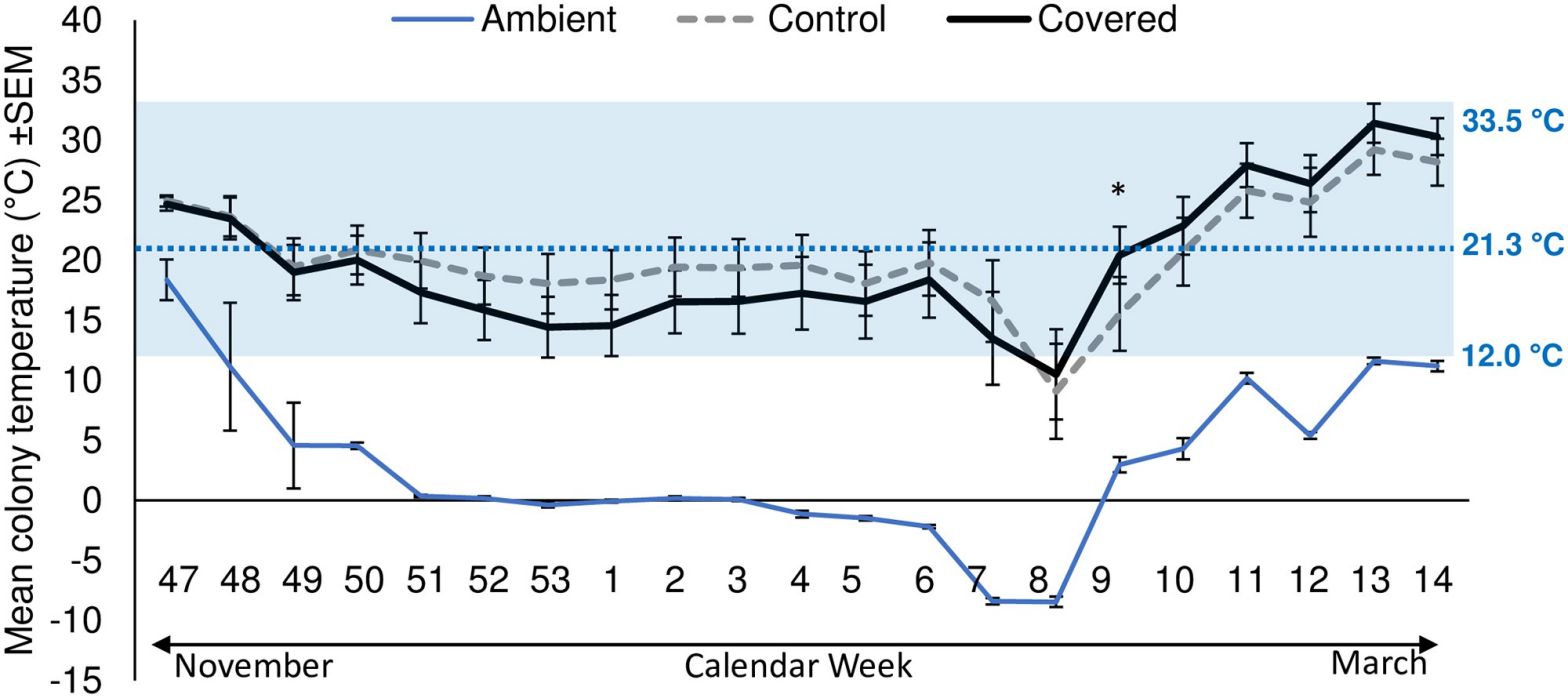
Average weekly temperature (±SEM) of colonies by treatment (control vs covered). Treatments were significantly warmer than the ambient temperature from within empty sentinel colonies, however, no difference in temperature was observed between covered and uncovered colonies. Throughout the winter colonies in both treatments were able to maintain cluster temperatures within the normal range for honey bee colonies (normal range 12–33.5°C indicated by the shaded box and the blue dotted line indicates the average overwintering cluster temperature for healthy overwintering colonies; [45].

All live colonies maintained significantly higher cluster temperatures than the ambient temperature measured inside of empty sentinel equipment (F_1, 42.77_ = 10.37, *p* = 0.002) and maintained temperatures within the range reported as normal for winter clusters (12–33.5°C; 41) (Fig 3). There was no significant difference in the overall temperature between covered and control colonies (F_1, 39.93_ = 0.01, *p* = 0.93). Temperature varied by week (F_20, 779_ = 21.30, *p* = <0.0001) and there was a significant interaction between treatments and calendar week (F_20, 779_ = 1.29, *p* = 0.005). Specifically, covered colonies maintained marginally warmer cluster temperatures compared to control colonies in the late winter/early spring period (i.e., calendar weeks 8,10,13–14, and maintained a significantly higher cluster temperature on week 9 (Fig 3; S5 Table).

**Fig 4:**
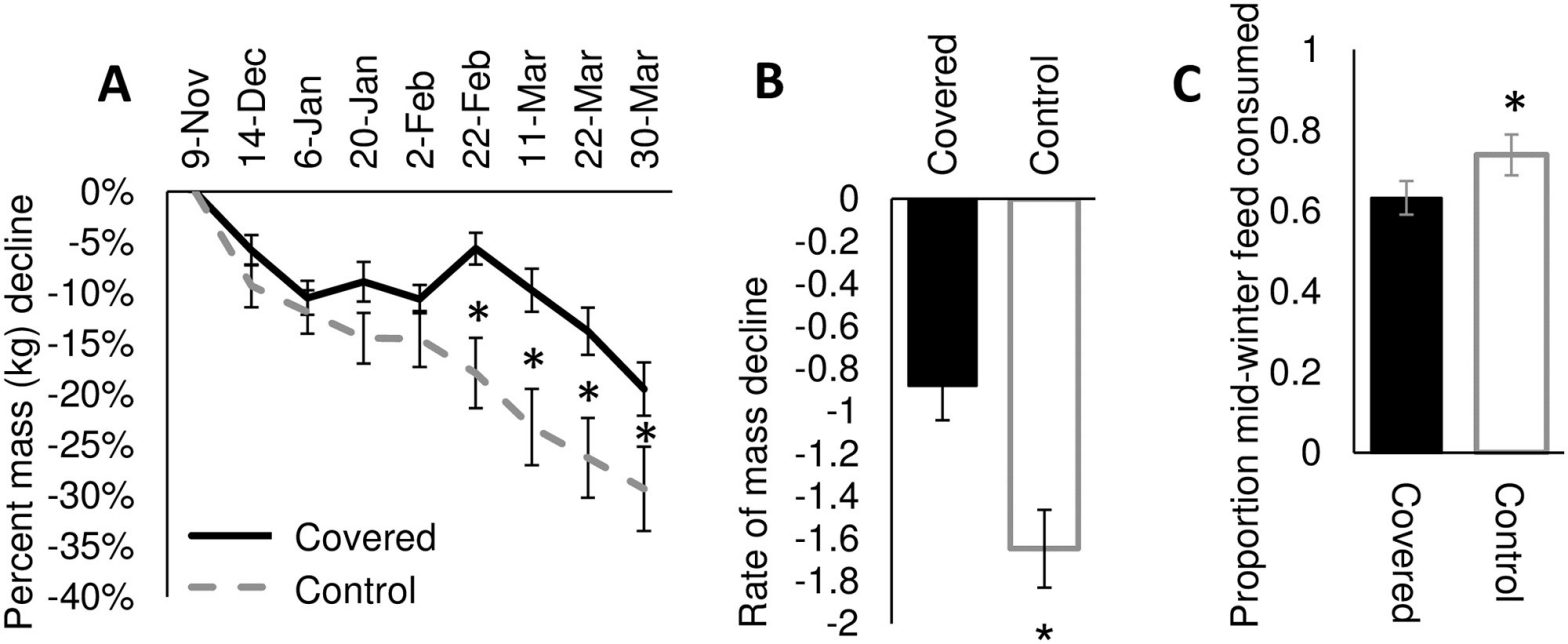
(A) Percent change in mass of colonies by treatment (covered vs control). Percent change is based on the starting mass in kg of colonies on November 9^th^, 2020. (B) Mean rate of mass decline (kg mass/sample period) in colonies by treatment. (C) Proportion of mid-winter supplemental granular sugar cake colonies consumed by treatment. Asterisks represent significance at α = 0.05 and error bars are one standard error of the mean.

Overall, covered colonies lost significantly less mass than the uncovered controls (F_1, 41.35_ = 6.91, *p* = 0.01). Mass decline varied by sample date (F_8, 309.7_ = 50.12, *p* = <0.0001) and a significant interaction of treatment and sample date occurred (F_8, 309.7_ = 6.65, *p* = <0.0001). Both hive treatments lost mass from the beginning of the overwintering period (November 9), with no significant difference in percent mass lost between treatments for the first eight weeks (Fig 4A; S6 Table). However, beginning at the February 22^nd^ measurement period, covered colonies lost significantly less mass than control colonies (Fig 4A; S6 Table). This trend continued through the rest of the experiment, ending on March 30 (Fig 4A; S6 Table). Over the season, control colonies declined in mass (kg loss per sample period) at a significantly greater (almost two-fold) rate than covered colonies (F_1, 41_ = 9.74, *p* = 0.003; Fig 4B). In addition to consuming more of their honey/sucrose food stores, control colonies also consumed significantly more of the solid granular sugar cake provided as mid-winter supplementation in February (F_1, 41.2_ = 4.59, *p* = 0.04; Fig 4C).

**Fig 5:**
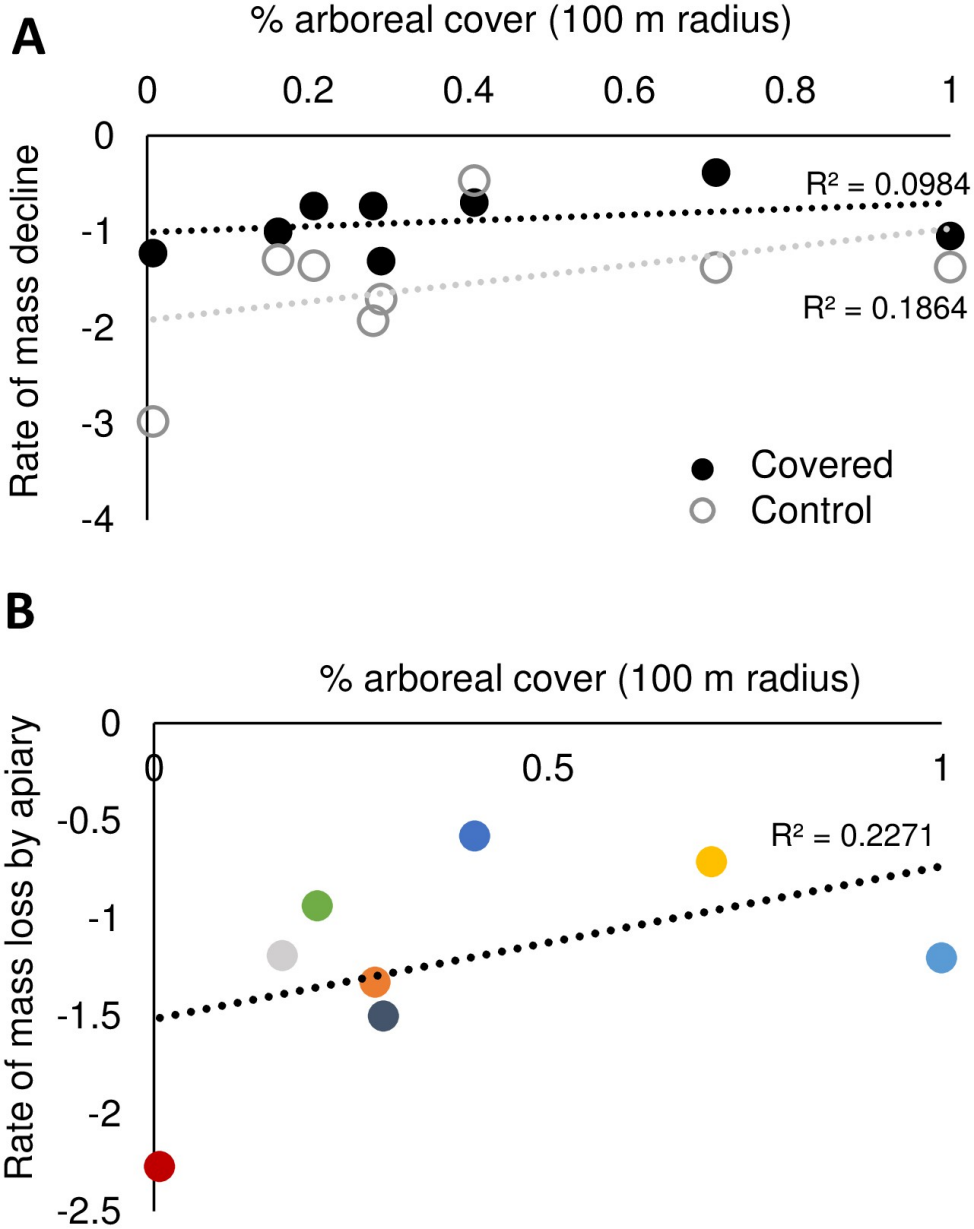
Relationship between the rate of mass decline in (A) covered (solid circles) and control (open circles) colonies and (B) colonies at the apiary level with the proportion of arboreal tree cover within 100 m of apiaries.

As in some previous studies [17, 39], landscape composition around each apiary was a significant predictor of mass declines, with woodland having a positive relationship with percent change in mass (F = 7.52, *p* = 0.006) and developed land being a significantly negative predictor (F = 15.16, *p* = 0.0001) (S7 Table). However, in both cases, the coefficient of determinations for these models is very low (<0.1; S7 Table), indicating that any landscape composition predictions are very weak. There were no significant relationships between covered or control colony rate of mass decline with the proportion arboreal tree cover within 100m of the apiary (F_1, 18_ = 0.04, *p* = 0.85; F_1, 20_ = 2.80, *p* = 0.11 for covered and control respectively; Fig 5A) or at the apiary level (F_1, 41_ = 2.07, *p* = 0.16; Fig 5B).

**Fig 6:**
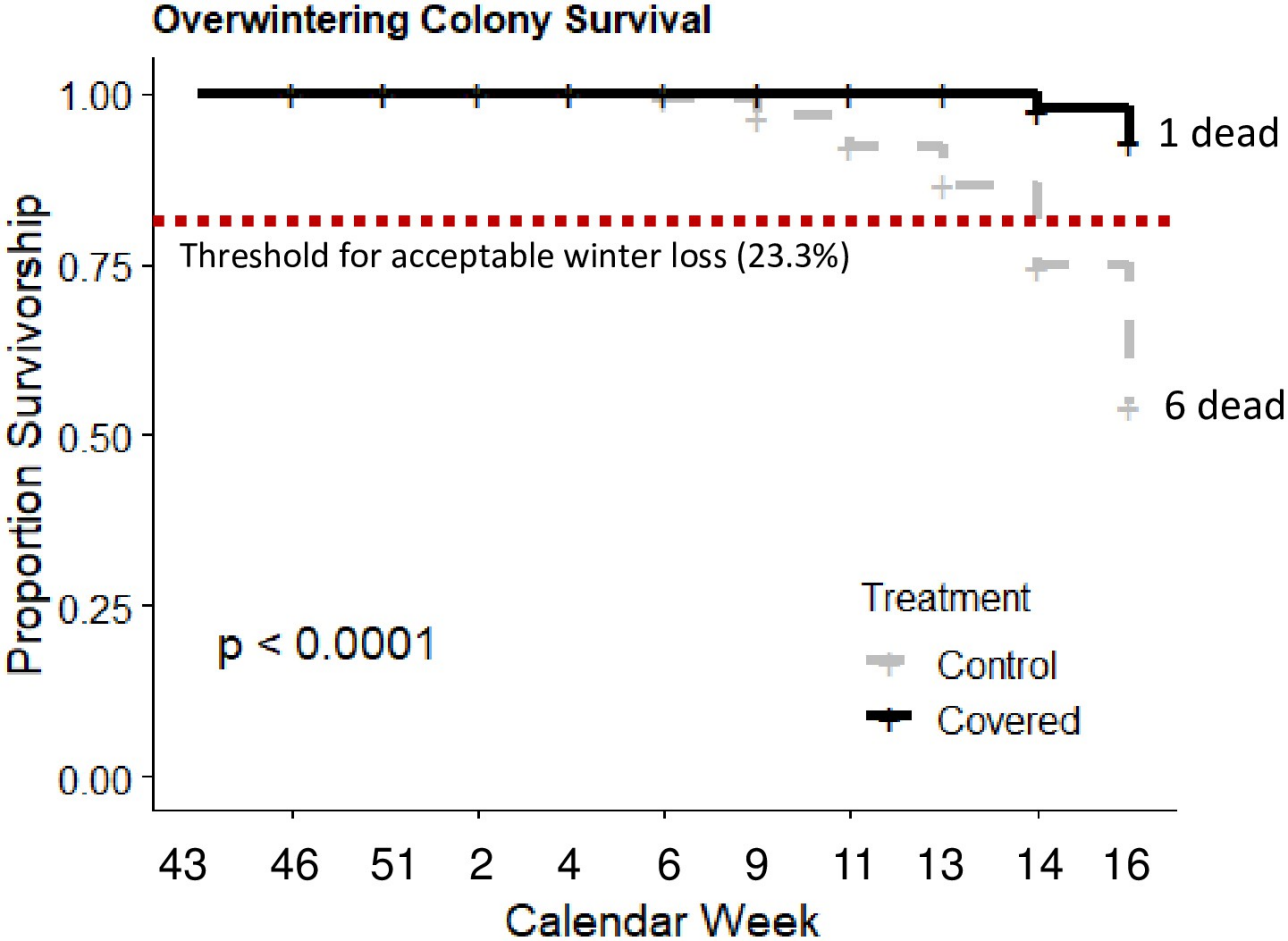
Proportion survivorship of colonies by treatment (covered vs control). Survivorship was significantly higher for colonies that were wrapped compared to those that were left unwrapped.

**Fig 7:**
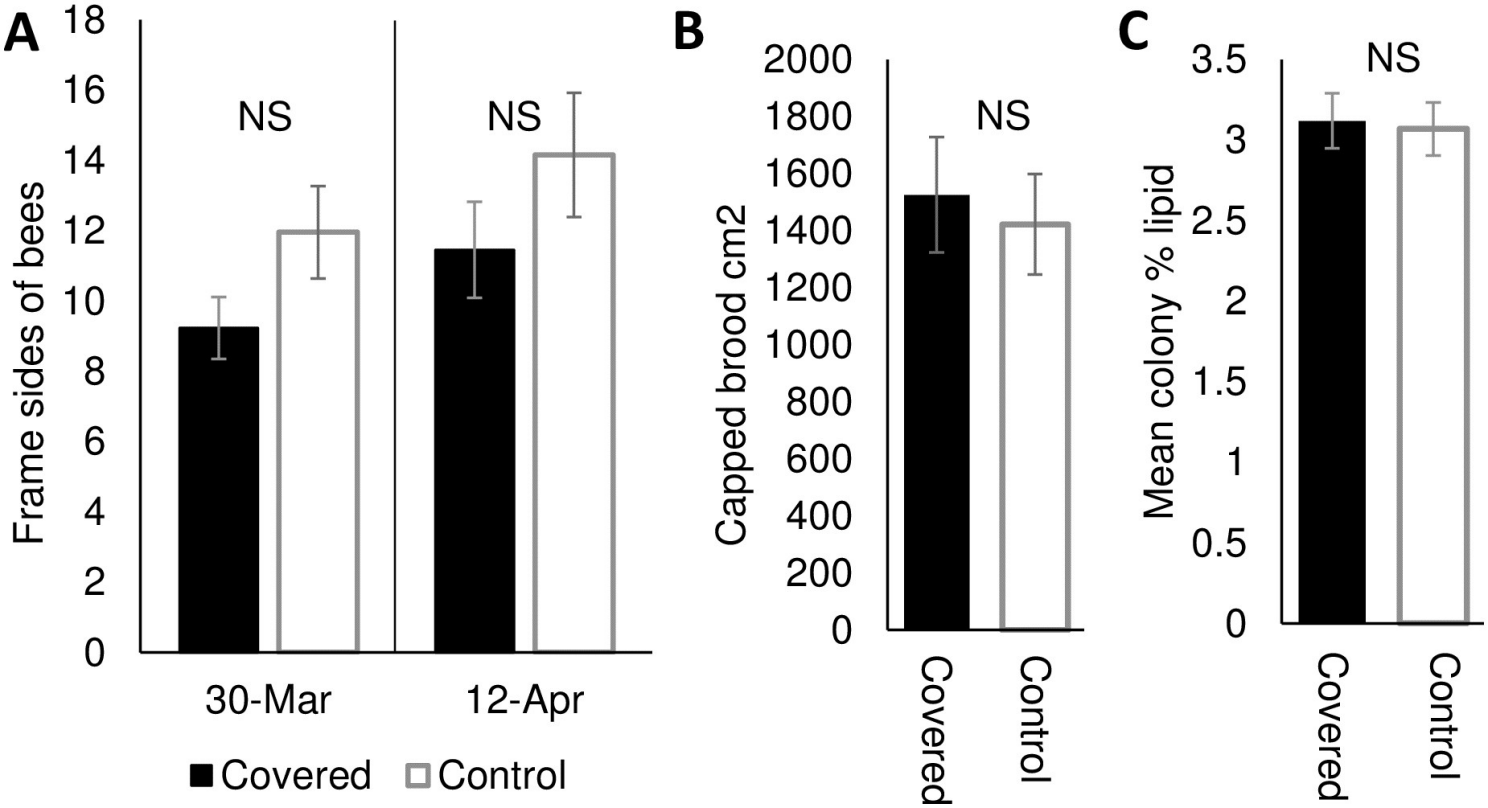
(A) Mean frames sides of bees in covered and control colonies on March 30^th^ and April 12^th^, 2021 (these dates are a representation of Spring build-up). (B) Mean capped brood area (cm^2^) and (C) mean percent lipid of individual bees within colonies in covered vs control treatments on March 30^th^, 2021.

Significantly more covered colonies survived the winter than control colonies (4.8% mortality in covered treatment; Z = 3.382, *p* = 0.0007; Fig 6), remaining above the previously reported threshold of acceptable winter losses of 23.3% [7], while control colony losses exceeded this number (27.3% mortality in control treatment). In colonies that survived the winter, adult bee populations at the end of the overwintering period did not significantly differ between treatment groups (March 30; F_1, 37_ = 0.85, *p* = 0.36 and April 12; F_1, 34_ = 1.60, *p* = 0.21; Fig 7A). Immature bee population (capped brood; F_1, 33_ = 0.13, *p* = 0.72; Fig 7B) and fat stores of bees collected from the interiors of each colony (F_1, 37_ = 0.04, *p* = 0.84; Fig 7C) at the end of overwintering (March 30^th^) also did not significantly differ.

## Discussion

We found that, across eight experimental apiaries, covering honey bee hives with corrugated polypropylene board and topping them with foam insulation decreased food store consumption and improved overwintering survival compared to identically managed hives without covers and insulation. We also observed covered hives maintained marginally increased cluster temperatures in the springtime compared to uncovered controls. Thus, we provide new and updated evidence for the efficacy of these types of hive coverings in a temperate agricultural landscape under modern small-scale beekeeping conditions.

Successful colony overwintering requires multiple conditions be met. First, disease and pest pressure must be controlled. The most detrimental of these, *Varroa destructor* mites, introduce viral pathogens [29], feed upon the fat body of their host [46], and are associated with reduced fat stores in pre-overwintering bees [47]. In our study, the ubiquitous pest was present and reached treatment thresholds before overwintering, but colonies were uniformly treated and Varroa controlled accordingly, leading to low mite levels that were not different between our treatment groups. Adequate nutritional resource availability is paramount for bees to properly thermoregulate through the winter [20, 38, 48]. Thus, beekeeping recommendations include ensuring honey stores are at or above a threshold necessary for a given climate [15, 23, 28]. For example, in the temperate climate of Pennsylvania, USA, colonies with at least 30kg of honey stores had a 95% chance of surviving the winter, while those with lower stores were more likely to perish [38]. Obtaining these stores can be accomplished by keeping bees in areas with high habitat suitability [31, 32, 48], and beekeepers often provide pre-overwintering colonies with artificial feed, such as sugar or high fructose corn syrup solutions or dry sugar cakes [14, 49]. Here, we ensured that all colonies entered the winter with appropriate food stores (S2 Fig) and provided a ‘mid-winter supplement’ of dry sugar mid-winter (S3 Fig).

While previous work has shown that the surrounding landscape can have significant effects on the foraging and food provisioning of honey bee colonies [31, 32, 48, 50–52], we found only very minor significant relationships between the surrounding landscape features of our apiaries and colony metrics; even the significant predictors (woodland and developed land) were poor predictors of success (low model r^2^; S7 Table). Unlike other studies that explicitly targeted specific land use profiles [17, 32, 47, 52], our study used existing apiaries that had been occupied by the University of Illinois Bee Research Facility for >5 years (in some cases >20 years), which were chosen for a mixture of perceived quality and accessibility. Thus, it may be that these apiaries were not different enough for these factors to come fully into play, though landscape composition did vary across apiaries (S5 Fig; S3 Table). Perhaps more importantly, all the colonies used in this experiment were provided with large quantities of fall supplemental feeding on top of existing food stores, which would normally not be done in an experiment evaluating land use [17]. That is, we used honey and sucrose syrup feed to bring all colonies, regardless of location, well above the minimum threshold for overwintering success (S2 Fig).

Thus, independent of the apiary location, covered hives performed significantly better than controls. All honey bee colonies form clusters during cold weather to conserve and efficiently produce heat. Previous work has shown that, during the spring and summer, brood nest temperatures average 35.5°C, fluctuating 1–2°C around this average; in the winter, the worker cluster maintains an average temperature of 21.3°C until warm temperatures return and brood rearing commences [45]. In our experiments, there was no overall difference in cluster temperature between treatments (Fig 3) consistent with foundations studies showing that bees can efficiently thermoregulate whether they are covered or not [20]. However, covered colonies were only marginally warmer beginning at calendar week 8 (approximately February 20^th^–24^th^), after which ambient (unoccupied) hive temperatures first rose above 0°C. Thus, the covers did not appear to affect temperature regulation during the coldest time in the winter; rather, only once temperatures rose and colony buildup began did changes trend towards warmer when covered. Because the temperatures of both treatments were within the reported normal range for either a winter cluster (sub-0°C ambient temperatures) or spring buildup (above freezing ambient temperatures; [45]), it is not clear if there was a benefit of maintaining these higher temperatures, especially given that we saw no difference in adult (frames of bees; Fig 7A) or immature (greater capped brood area; Fig 7B) population during March or April, nor did we detect differences in fat stores of presumed nurse bees from these time points (Fig 7C).

Despite their similar or elevated temperature levels, covered colonies consumed significantly less of their food stores than uncovered controls. The pattern of mass decline parallels temperature; for the season, covered colonies declined in mass at a significantly lower rate than controls. However, this again appears primarily driven by differences beginning the last week of February (2021 calendar week 8) and increasing as ambient temperatures rose above freezing, i.e., during spring colony buildup. Consumption of the solid granular sugar feed, added on Feb 2 (calendar week 6), matches this pattern, with control colonies consuming significantly more of the food source from its addition through March 30 (calendar week 14). Thus, covered colonies were able to maintain normal thermoregulatory temperatures, while consuming significantly less stored food, suggesting hive covers may reduce the energetic cost of nest thermoregulation.

At the operational level (i.e., all colonies included in study), colony losses were rather low (16.3%) compared to the state of Illinois 2020–21 average of 47% losses [7]. Covered colonies experienced significantly lower mortality throughout the course of the experiment compared to uncovered controls, with only a single colony perishing (Fig 6). Control colonies experienced 28.6% mortality, with the death of 6 total colonies (Fig 6); this rate is similar to the 2020–21 national total winter loss (33%; [7]). While it is lower than the state average (47%), it is still higher than the 2020–21 surveyed acceptable loss percentage (23.3%; [7]). Thus, covering and insulation significantly reduced colony losses and brought operational losses into the “acceptable loss” range. We note, however, that we did not perform any economic analyses to assess the cost of covering (in materials and labor) vs the value of greater overwintering success; such a comparison will likely be important for stakeholders as they weigh the benefits of different inputs for overwintering preparation.

While it may seem surprising that coverings would have an effect in an area without extremely cold winters, hive construction material has also been shown to affect internal hive environment at a similar latitude [36], with polyurethane hives reducing temperature fluctuations and improving humidity stability. Therefore, coverings or construction material may buffer colonies from temperature or weather fluctuations, helping them maintain colony homeostasis more efficiently. As we face a future of shifting temperature and weather norms that come with a changing climate [53], finding management strategies that can provide honey bee colonies with better stress tolerance will be critical to maintaining sustainable pollinator management.

## Supporting information

S1 Fig (A) Pre-overwintering Varroa mite levels across apiaries during the week of October 5^th^-8^th^ 2020. Mite levels were not checked at the Perkins or Quarry apiaries. Mean mites loads across all sites were 10.54 mites per colony. (B) Pre-overwintering mite levels from a subset of colonies that were rechecked on November 9^th^, 2020, after a treatment by oxalic acid vaporization on October 12^th^. Mean mites loads were successfully reduced to 2.20 mites per colony (below the 1% threshold of 3 mites per 300 bees sampled) and were significantly lower compared to the October sample (T_48_ = 2.99, P = 0.004).
(PDF)

S2 Fig Mean pre-overwintering (November 9^th^, 2020) mass of colonies by apiary. Overall mass varied significantly by apiary (F_7,34_ = 2.56, p = 0.03). Letters represent the Tukey HSD post-hoc differences in least squared means comparisons for mean mass by apiary; p<0.05. Red dotted line is the suggested weight threshold (30 kg) to enter the winter with a >95% expected survival rate (Döke et al. 2019). All apiaries had colonies above the minimum threshold for expected winter survival (T_7_ = 15.42, P<0.0001). There were no differences between the colonies that would later form the wrapped and unwrapped treatment groups (T_14_ = 1.40, *p* = 0.18).
(PDF)

S3 Fig (A) 1-inch (2.54 cm) shim lined with 27 gauge 1/8^th^ inch (0.32 cm) wire mesh hardware cloth placed at the top of the colony above the honey super. (B) Sugar cake patty that consisted of 7 lbs. (3.18 kg) dry granulated white sugar mixed with 1 cup (236.59 milliliters) of water added on top of shim.
(PDF)

S4 Fig A comparison of weight in 42 colonies that were measured via classic methods using a postal scale as in Dolezal et al. 2019 (17) and then measured using the tilt crane scale method. The two methods of measuring colony mass are highly significantly correlated (F_1, 40_ = 136.7, *p* = <0.0001).
(PDF)

S5 Fig Proportion of land cover surrounding each apiary within a 1 km radius. (PDF)

S1 Table Trade Journal Publication Investigating Hive Cover Effectiveness and/or Experimental Overwintering Success. (PDF)

S2 Table Number of colonies in each treatment across the eight research apiaries studied in winter 2020–2021. (PDF)

S3 Table Surrounding land use features of apiaries within a 1-km radius during the winter 2020–2021. (PDF)

S4 Table Pearson’s correlation of the land use features surrounding apiaries within a 1-km radius during the winter of 2020–2021. (PDF)

S5 Table Least squared means comparison of colony temperature across calendar weeks in control and covered colonies. (PDF)

S6 Table Simple Effect Comparisons of Treatment (wrapped vs unwrapped) by date Adjustment for Multiple Comparisons: Tukey-Kramer. (PDF)

S7 Table Multiple regression for percent change in colony mass and the rate of decline in colony mass across the apiaries using landscape features cropland, woodland, grassland, and developed land as possible parameters. Stepwise model selection was used to obtain the final variables in each model (P<0.15 for inclusion in the model).
(PDF)
