# Supplementary figures and images for: Honey bee hive covers reduce food consumption and colony mortality during overwintering
Source: PLoS One. 2022 Apr 4;17(4):e0266219. doi: 10.1371/journal.pone.0266219 (PMC8979464; doi:10.1371/journal.pone.0266219)

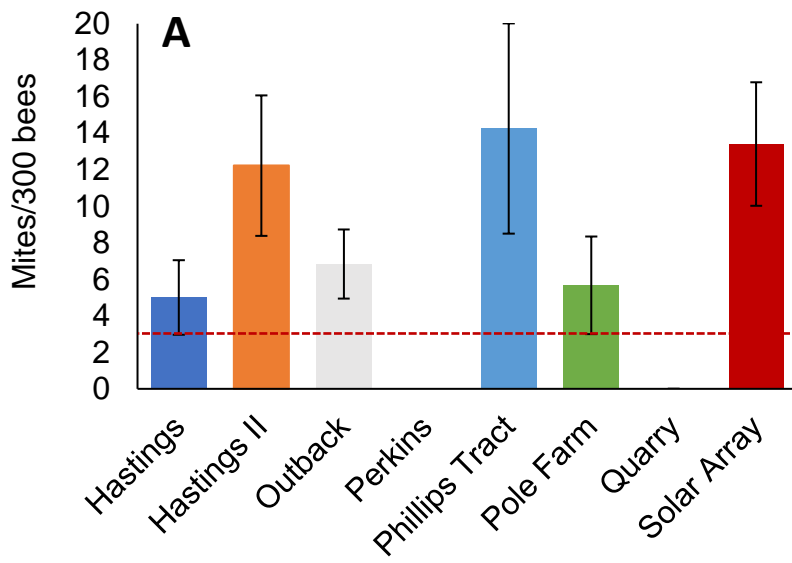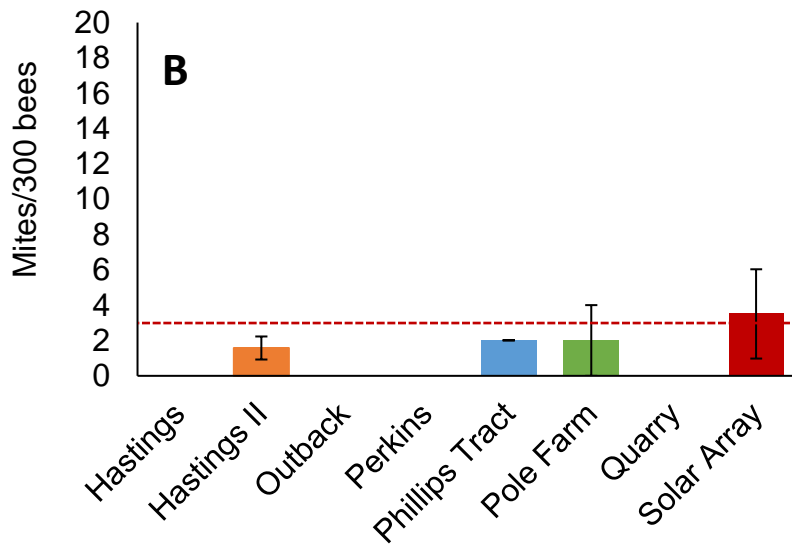

Supplement: S1 Fig — (A) Pre-overwintering Varroa mite levels across apiaries during the week of October 5th-8th 2020. Mite levels were not checked at the Perkins or Quarry apiaries. Mean mites loads across all sites were 10.54 mites per colony. (B) Pre-overwintering mite levels from a subset of colonies that were rechecked on November 9th, 2020, after a treatment by oxalic acid vaporization on October 12th. Mean mites loads were successfully reduced to 2.20 mites per colony (below the 1% threshold of 3 mites per 300 bees sampled) and were significantly lower compared to the October sample (T48 = 2.99, P = 0.004). (PDF) [file pone.0266219.s001.pdf]

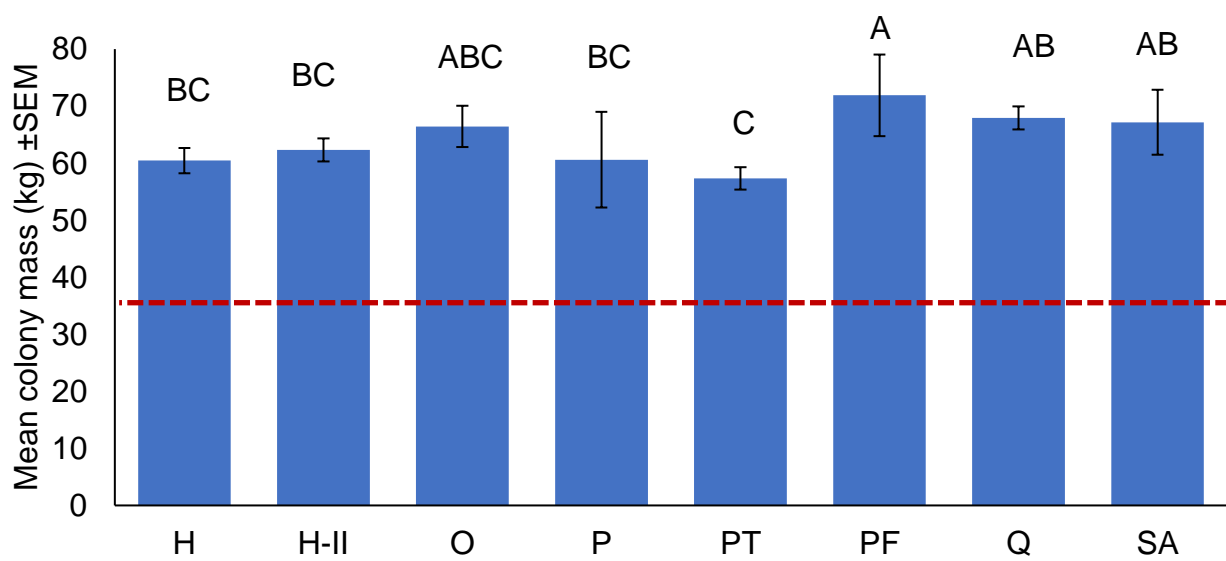

Supplement: S2 Fig — Overall mass varied significantly by apiary (F7,34 = 2.56, p = 0.03). Letters represent the Tukey HSD post-hoc differences in least squared means comparisons for mean mass by apiary; p<0.05. Red dotted line is the suggested weight threshold (30 kg) to enter the winter with a >95% expected survival rate (Döke et al. 2019). All apiaries had colonies above the minimum threshold for expected winter survival (T7 = 15.42, P<0.0001). There were no differences between the colonies that would later form the wrapped and unwrapped treatment groups (T14 = 1.40, p = 0.18). (PDF) [file pone.0266219.s002.pdf]

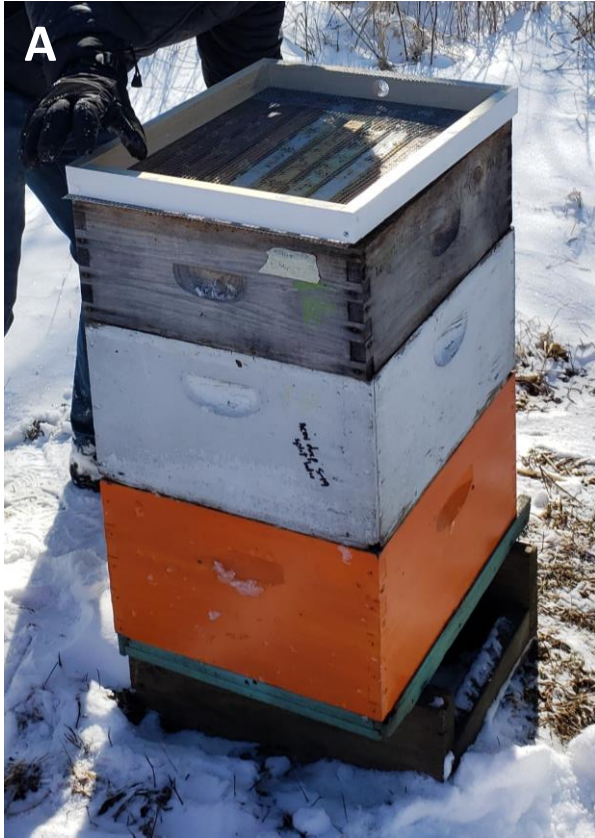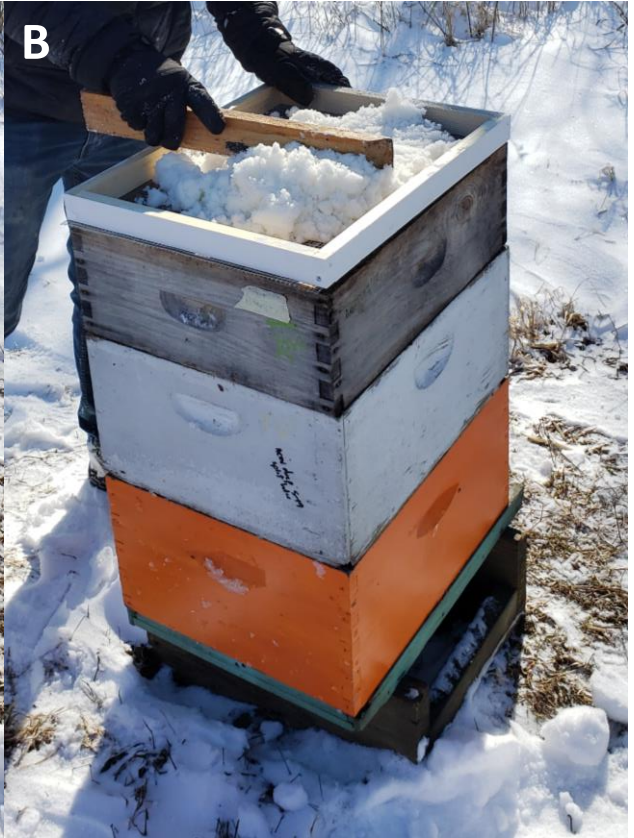

Supplement: S3 Fig — (A) 1-inch (2.54 cm) shim lined with 27 gauge 1/8th inch (0.32 cm) wire mesh hardware cloth placed at the top of the colony above the honey super. (B) Sugar cake patty that consisted of 7 lbs. (3.18 kg) dry granulated white sugar mixed with 1 cup (236.59 milliliters) of water added on top of shim. (PDF) [file pone.0266219.s003.pdf]

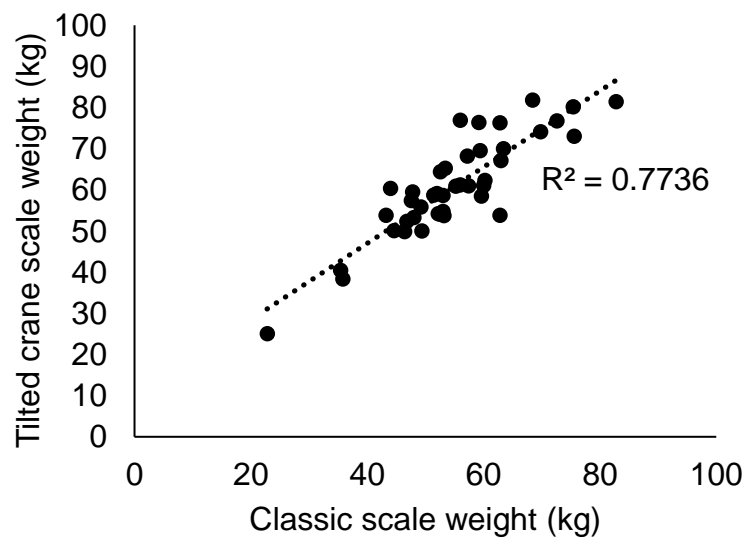

Supplement: S4 Fig — The two methods of measuring colony mass are highly significantly correlated (F1, 40 = 136.7, p = <0.0001). (PDF) [file pone.0266219.s004.pdf]

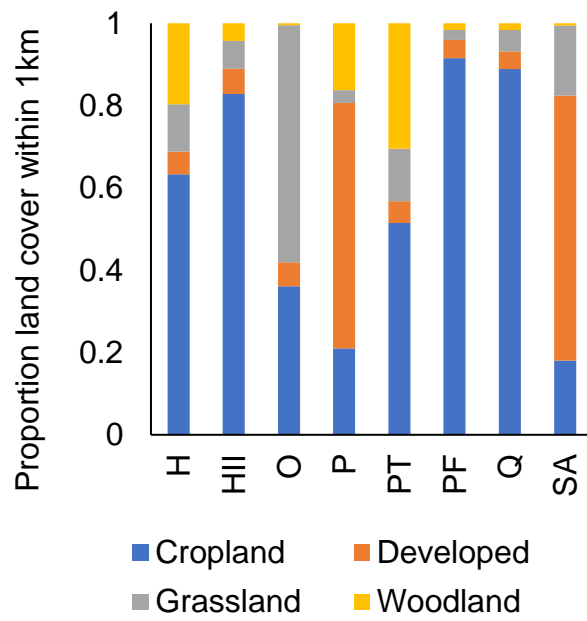

Supplement: S5 Fig — (PDF) [file pone.0266219.s005.pdf]
