# Supplementary material for: Honey bee hive covers reduce food consumption and colony mortality during overwintering
Source: PLoS One. 2022 Apr 4;17(4):e0266219. doi: 10.1371/journal.pone.0266219 (PMC8979464; doi:10.1371/journal.pone.0266219)
Supplement: S1 Table — (PDF) [file pone.0266219.s006.pdf]

Braun, E. 1934. Twelve years' experimental results of wintering in Manitoba. American Bee Journal. 74:492-501.

Jager, F. 1923. Cellar wintering of bees. American Bee Journal. 63:491-492.

Miller, A.C. 1901. Tarred paper for packing. American Bee Journal. 41:718.

Miller, A.C. 1903. Tarred paper for winter protection. Gleanings in Bee Culture. 31:371.

Szabo, T.I. 1989a. Thermology of wintering honev-bee colonies in 4-colonv packs. 1. The direct effects of hive insulation on colony temperatures. *American Bee Journal*. 129:338-339.

Szabo, T.I. 1989b. Thermology of wintering honev-bee colonies in 4-colonv packs. 2. Effects of the removal of insulation on colony temperatures. *American Bee Journal*. 129:405-406.

Szabo, T.I. 1989c. Thermology of wintering honev-bee colonies in 4-colonv packs. 3. Cluster Positions. *American Bee Journal*. 129:472-473.

Szabo, T.I. 1993. Brood rearing in outdoor wintered colonies. *American Bee Journal*. 133:723-724.
