# Supplementary material for: Honey bee hive covers reduce food consumption and colony mortality during overwintering
Source: PLoS One. 2022 Apr 4;17(4):e0266219. doi: 10.1371/journal.pone.0266219 (PMC8979464; doi:10.1371/journal.pone.0266219)
Supplement: S2 Table — (PDF) [file pone.0266219.s007.pdf]

Site	Treatment		Total
	Covered	Uncovered	
H	2	2	4
HII	5	5	10
O	2	4	6
P	2	1	3
PT	4	4	8
PF	2	1	3
Q	2	2	4
SA	2	3	5
**Total**	21	22	43
