# Supplementary material for: Honey bee hive covers reduce food consumption and colony mortality during overwintering
Source: PLoS One. 2022 Apr 4;17(4):e0266219. doi: 10.1371/journal.pone.0266219 (PMC8979464; doi:10.1371/journal.pone.0266219)
Supplement: S3 Table — (PDF) [file pone.0266219.s008.pdf]

Land Use Type	O	H	HII	Q	PT	PF	P	SA
Corn	22.90%	52.54%	30.55%	33.83%	25.01%	47.10%	13.17%	7.07%
Soybeans	9.57%	10.61%	52.11%	55.04%	24.90%	44.24%	7.79%	10.50%
Winter Wheat	0.43%	0.00%	0.06%	0.03%	0.72%	0.00%	0.00%	0.03%
Dbl Crop WinWht/Soybeans	0.14%	0.06%	0.06%	0.00%	0.09%	0.00%	0.00%	0.20%
Alfalfa	3.04%	0.11%	0.09%	0.00%	0.77%	0.20%	0.03%	0.26%
Other Hay/Non Alfalfa	6.38%	0.63%	0.23%	0.26%	0.57%	0.17%	0.29%	1.20%
Clover/Wildflowers	0.00%	0.00%	0.03%	0.00%	0.00%	0.03%	0.00%	0.00%
Sod/Grass Seed	1.74%	0.00%	0.00%	0.00%	0.00%	0.00%	0.00%	0.31%
Fallow/Idle Cropland	0.14%	0.32%	0.11%	0.80%	2.52%	0.43%	0.03%	1.66%
Open Water	0.87%	0.06%	0.00%	0.06%	0.09%	0.00%	1.12%	0.72%
Developed/Open Space	1.16%	3.76%	5.25%	2.20%	2.12%	3.56%	24.51%	6.12%
Developed/Low Intensity	2.32%	0.86%	0.66%	0.80%	2.58%	0.89%	28.98%	33.17%
Developed/Med Intensity	1.16%	0.40%	0.09%	0.46%	0.37%	0.03%	4.78%	19.89%
Developed/High Intensity	0.14%	0.14%	0.00%	0.72%	0.00%	0.00%	0.26%	4.32%
Barren	0.14%	0.29%	0.14%	0.03%	0.14%	0.00%	0.03%	0.14%
Deciduous Forest	0.43%	6.05%	2.44%	0.72%	13.38%	0.95%	4.38%	0.34%
Evergreen Forest	0.00%	0.00%	0.00%	0.00%	0.00%	0.00%	0.00%	0.00%
Mixed Forest	0.00%	5.99%	1.20%	0.86%	13.58%	0.63%	9.59%	0.17%
Shrubland	0.00%	0.00%	0.00%	0.00%	0.57%	0.00%	0.00%	0.03%
Grassland/Pasture	49.42%	10.55%	6.39%	4.15%	9.63%	1.78%	2.78%	13.85%
Woody Wetlands	0.00%	7.54%	0.60%	0.06%	2.95%	0.00%	2.21%	0.00%
Herbaceous Wetlands	0.00%	0.09%	0.00%	0.00%	0.00%	0.00%	0.06%	0.00%
