# Supplementary material for: Honey bee hive covers reduce food consumption and colony mortality during overwintering
Source: PLoS One. 2022 Apr 4;17(4):e0266219. doi: 10.1371/journal.pone.0266219 (PMC8979464; doi:10.1371/journal.pone.0266219)
Supplement: S4 Table — (PDF) [file pone.0266219.s009.pdf]

Pearson Correlation Coefficients, N = 8				
	**Cropland**	**Woodland**	**Developed**	**Grassland**
**Cropland**	1	-0.15858	-0.76523	-0.42012
**Woodland**	-0.15858	1	-0.05769	-0.24894
**Developed**	-0.76523	-0.05769	1	-0.14508
**Grassland**	-0.42012	-0.24894	-0.14508	1
