# Supplementary material for: Honey bee hive covers reduce food consumption and colony mortality during overwintering
Source: PLoS One. 2022 Apr 4;17(4):e0266219. doi: 10.1371/journal.pone.0266219 (PMC8979464; doi:10.1371/journal.pone.0266219)
Supplement: S5 Table — (PDF) [file pone.0266219.s010.pdf]

Calendar week	Treatment	Treatment	Estimate	SE	DF	t Value	Pr > |t|	Adj P
1	Control	Covered	0.04286	0.06759	804	0.63	0.5262	0.5262
2	Control	Covered	0.02028	0.06759	804	0.30	0.7642	0.7642
3	Control	Covered	0.02175	0.06759	804	0.32	0.7477	0.7477
4	Control	Covered	0.01788	0.06759	804	0.26	0.7915	0.7915
5	Control	Covered	-0.00245	0.06759	804	-0.04	0.9711	0.9711
6	Control	Covered	-0.00693	0.06759	804	-0.10	0.9183	0.9183
7	Control	Covered	0.05866	0.06853	804.3	0.86	0.3923	0.3923
8	Control	Covered	-0.1137	0.06853	804.3	-1.66	0.0976	0.0976
9	Control	Covered	-0.2086	0.06853	804.3	-3.04	0.0024	**0.0024**
10	Control	Covered	-0.1161	0.07031	803.6	-1.65	0.0991	0.0991
11	Control	Covered	-0.1089	0.07031	803.6	-1.55	0.1219	0.1219
12	Control	Covered	-0.1137	0.07129	803	-1.59	0.1111	0.1112
13	Control	Covered	-0.1210	0.07129	803	-1.70	0.0899	0.0899
14	Control	Covered	-0.1194	0.07129	803	-1.67	0.0945	0.0945
47	Control	Covered	-0.05087	0.06759	804	-0.75	0.4519	0.4519
48	Control	Covered	-0.05239	0.06759	804	-0.78	0.4385	0.4385
49	Control	Covered	-0.04815	0.06759	804	-0.71	0.4764	0.4764
50	Control	Covered	-0.03857	0.06759	804	-0.57	0.5684	0.5684
51	Control	Covered	0.01263	0.06759	804	0.19	0.8518	0.8518
52	Control	Covered	0.01658	0.06759	804	0.25	0.8063	0.8063
53	Control	Covered	0.03771	0.06759	804	0.56	0.5771	0.5771
