# Supplementary material for: Honey bee hive covers reduce food consumption and colony mortality during overwintering
Source: PLoS One. 2022 Apr 4;17(4):e0266219. doi: 10.1371/journal.pone.0266219 (PMC8979464; doi:10.1371/journal.pone.0266219)
Supplement: S6 Table — (PDF) [file pone.0266219.s011.pdf]

Date	Treatment	Treatment	Estimate	SE	DF	t Value	Pr > |t|	Adj P
Nov. 9	**Control**	**Covered**	9.73E-15	0.03135	87.05	0.00	1.0000	1.0000
Dec 14	**Control**	**Covered**	-0.02967	0.03135	87.05	-0.95	0.3465	0.3447
Jan 6	**Control**	**Covered**	-0.01322	0.03135	87.05	-0.42	0.6742	0.6734
Jan 20	**Control**	**Covered**	-0.05575	0.03135	87.05	-1.78	0.0788	0.0763
Feb 2	**Control**	**Covered**	-0.03715	0.03151	88.63	-1.18	0.2415	0.2392
Feb 22	**Control**	**Covered**	-0.1210	0.03187	92.23	-3.80	0.0003	**0.0002**
Mar 11	**Control**	**Covered**	-0.1307	0.03207	94.31	-4.08	<.0001	**<.0001**
Mar 22	**Control**	**Covered**	-0.1177	0.03207	94.31	-3.67	0.0004	**0.0003**
Mar 30	**Control**	**Covered**	-0.1058	0.03269	100.8	-3.24	0.0016	**0.0013**
