# Supplementary material for: Honey bee hive covers reduce food consumption and colony mortality during overwintering
Source: PLoS One. 2022 Apr 4;17(4):e0266219. doi: 10.1371/journal.pone.0266219 (PMC8979464; doi:10.1371/journal.pone.0266219)
Supplement: S7 Table — Stepwise model selection was used to obtain the final variables in each model (P<0.15 for inclusion in the model). (PDF) [file pone.0266219.s012.pdf]

Factor	Land-use Variable	Estimate	SE	F value	P value	Model R-Square
% Change in Mass	Woodland	0.15430	0.056	7.52	**0.0064**	0.0495
	Developed	-0.11155	0.029	15.16	**0.0001**	0.0687
Rate of decline	Developed	-0.95404	0.595	2.57	0.1165	0.0590
